# Diabetes Status, c-Reactive Protein, and Insulin Resistance in Community-Acquired Pneumonia—A Prospective Cohort Study

**DOI:** 10.3390/jcm13010245

**Published:** 2023-12-31

**Authors:** Arnold Matovu Dungu, Camilla Koch Ryrsø, Maria Hein Hegelund, Andreas Vestergaard Jensen, Peter Lommer Kristensen, Rikke Krogh-Madsen, Christian Ritz, Daniel Faurholt-Jepsen, Birgitte Lindegaard

**Affiliations:** 1 Department of Pulmonary and Infectious Diseases, Copenhagen University Hospital—North Zealand, 3400 Hillerød, Denmarkandreas.vestergaard.jensen.01@regionh.dk (A.V.J.); birgitte.lindegaard.madsen@regionh.dk (B.L.); 2 Centre for Physical Activity Research, Copenhagen University Hospital, Rigshospitalet, 2100 Copenhagen, Denmark; 3 Department of Endocrinology and Nephrology, Copenhagen University Hospital—North Zealand, 3400 Hillerød, Denmark; 4 Department of Clinical Medicine, Faculty of Health and Medical Sciences, University of Copenhagen, 2200 Copenhagen, Denmark; 5 Department of Infectious Diseases, Copenhagen University Hospital, 2650 Hvidovre, Denmark; 6 National Institute of Public Health, University of Southern Denmark, 1455 Copenhagen, Denmark; 7 Department of Infectious Diseases, Copenhagen University Hospital, Rigshospitalet, 2100 Copenhagen, Denmark

**Keywords:** community-acquired pneumonia, c-reactive protein, diabetes mellitus, chronic hyperglycaemia, acute hyperglycaemia, acute-on-chronic hyperglycaemia, insulin resistance

## Abstract

C-reactive protein (CRP) is commonly used to guide community-acquired pneumonia (CAP) treatment. A positive association between admission glucose and CRP levels has been observed in patients with CAP. The associations between prediabetes, unknown diabetes, acute-on-chronic hyperglycaemia, and CRP levels, and between admission CRP levels and insulin resistance (IR) in CAP, remain unexplored. This study investigated the associations firstly between chronic, acute, and acute-on-chronic hyperglycaemia and CRP levels, and secondly between admission CRP levels and IR in CAP. In a prospective cohort study of adults with CAP, the associations between chronic, acute, and acute-on-chronic hyperglycaemia (admission glucose minus HbA1c-derived average glucose) and CRP levels until admission day 3 were modelled with repeated-measures linear mixed models. IR was estimated with the homeostasis model assessment of IR (HOMA-IR). The association between admission CRP levels and HOMA-IR was modelled with linear regression. In 540 patients, no association between chronic, acute, or acute-on-chronic hyperglycaemia and CRP levels was found. In 266 patients, every 50 mg/L increase in admission CRP was associated with a 7% (95% CI 1–14%) higher HOMA-IR. In conclusion, our findings imply that hyperglycaemia does not influence CRP levels in patients with CAP, although admission CRP levels were positively associated with IR.

## 1. Introduction

Community-acquired pneumonia (CAP) remains a leading cause of hospitalisation and mortality across the globe [1,2]. Among the unmet needs in CAP research is identifying a biomarker to identify severe disease, guide treatment, and have a prognostic value [3]. C-reactive protein (CRP), an acute-phase reactant produced in the liver in response to the pro-inflammatory cytokine interleukin (IL)-6, is one of the most widely used biomarkers to guide clinical decisions in patients hospitalised with CAP [3,4,5]. A CRP decline of <50% within 3 days of admission has been associated with an increased risk of 30-day mortality in patients with CAP [6]. However, CRP is a non-specific biomarker of inflammation and is elevated in many chronic diseases typically seen in CAP cohorts, such as diabetes mellitus (DM) and advanced cancer [5,7,8,9]. Therefore, understanding which parameters affect CRP levels during CAP is of potential clinical significance.

States of chronic hyperglycaemia, such as prediabetes or DM, and acute hyperglycaemia (i.e., elevated plasma (p)-glucose levels) at admission are common in patients with CAP [10,11]. Furthermore, some individuals with prediabetes or DM have elevated CRP levels even in the absence of infection [9]. However, data on the impact of chronic and acute hyperglycaemia on inflammation in patients with non-coronavirus disease 2019 (COVID-19) CAP is sparse, as only 3 studies exist [12,13,14]. 

To date, no studies have investigated the association between other states of chronic hyperglycaemia, such as prediabetes and undiagnosed DM, and acute-on-chronic hyperglycaemia and CRP levels during admission in CAP. Identifying how hyperglycaemia affects CRP levels could significantly enhance the management of CAP by improving risk stratification and guiding treatment decisions based on a patient’s glycaemic status. Furthermore, acute hyperglycaemia during CAP is thought to be due to insulin resistance secondary to the acute stress response and high-grade inflammation [15]. However, the association between inflammation (measured with CRP) and insulin resistance has not been evaluated in patients with CAP.

We hypothesised that chronic, acute, and acute-on-chronic hyperglycaemia might impact CRP levels during hospitalisation and that high CRP levels at admission are associated with increased insulin resistance in patients with CAP. Therefore, our objectives were to explore the association between chronic, acute, and acute-on-chronic hyperglycaemia and CRP levels during hospitalisation and the association between CRP levels at admission and insulin resistance in patients with non-COVID-19 CAP.

## 2. Materials and Methods

### 2.1. Study Design

The present study is a sub-study of an ongoing prospective, observational cohort study of patients hospitalised with CAP, the Surviving Pneumonia Study cohort, conducted at Copenhagen University Hospital—North Zealand, in Denmark. Patients included in this sub-study were enrolled between January 2019 and January 2022, with a follow-up period of 3 days after admission. Inclusion criteria were adults aged 18 years or older with a new-onset infiltrate on a chest X-ray or computed tomography scan and presenting symptoms or clinical signs consistent with pneumonia, such as cough, sputum production, fever, hypothermia, chest pain, or abnormal chest auscultation. 

Patients diagnosed with COVID-19 or those without an HbA1c measurement during hospitalisation were excluded. The main reason for excluding patients with COVID-19 was that the study protocol was designed before the COVID-19 pandemic. Additionally, this study was intended to investigate CAP in a typical endemic setting, as outlined in the original protocol. Moreover, some patients at our institution were participating in international, double-blinded, randomised controlled trials investigating COVID-19 treatments, which at that time had an uncertain effect on the CRP response. Importantly, while HOMA-IR was a variable of interest in our analyses, the absence of data necessary to calculate HOMA-IR, as detailed below, did not exclude patients from the overall enrolment in the present study—only from specific analyses where HOMA-IR was the dependent outcome variable. 

The Scientific Ethics Committee at the Capital Region of Denmark (H-18024256) approved this study. Enrolment occurred within 24 h of admission after obtaining written informed consent. Written informed consent for patients deemed incapacitated at enrolment was obtained from their legal guardian or next of kin and an independent physician not affiliated with the study according to the guidelines of the Ethics Committee at the Capital Region of Denmark.

### 2.2. Data Collection

Trained personnel collected the demographic, medical history, clinical, laboratory, and microbiological data on standardised forms through a structured interview at study enrolment and from the medical record. Body mass index (BMI) was calculated from self-reported height and weight measured on an electric scale (Seca, Hamburg, Germany) within 48 h of admission. The Charlson comorbidity index, which assigns a weight to 19 specific comorbidities, was used to assess the comorbidity burden [16]. Disease severity was classified using the CURB-65 score, which assigns 1 point for the presence of confusion, urea > 7 mmol/L, respiratory rate ≥ 30/minute, blood pressure (either systolic ≤ 90 mmHg or diastolic ≤ 60 mmHg), and age 65 years or older, respectively [17]. Accordingly, the CURB-65 score was used to classify disease severity as mild (0–1), moderate (2), and severe (3–5) CAP [17]. The data were entered and stored in a REDCap database [18].

### 2.3. Laboratory Procedures

#### 2.3.1. Inflammation

Measurements of CRP in mg/L were performed at admission and the following 3 days during admission on the Dimension Vista^®^ 1500 platform (Siemens Healthineers, Erlangen, Germany). The peak CRP level was defined as the highest CRP level measured at any point from admission to day 3.

#### 2.3.2. Glucose Profile and Insulin

HbA1c in mmol/L was measured within 24 h of enrolment on the Tosoh G8 platform (Sysmex Corporation, Kobe, Japan). P-glucose in mmol/L was measured at admission while fasting p-glucose and p-insulin in pmol/L were measured between 7:30 a.m. and 8:30 a.m. on the day after study enrolment after an overnight fast from 7:30 p.m. P-glucose was measured on the Dimension Vista^®^ 1500 platform (Siemens Healthineers, Erlangen, Germany) and p-insulin on the Cobas e801 platform (Roche Diagnostics, Basel, Switzerland). Samples of fasting p-glucose and p-insulin from patients who received insulin during the fasting period were considered invalid.

#### 2.3.3. Insulin Resistance 

Insulin resistance was estimated with the original homeostasis model assessment of insulin resistance (HOMA-IR) based on fasting p-glucose and p-insulin with the formula: HOMA1-IR = fasting glucose (mmol/L) × fasting insulin (pmol/L)/135 [19,20]. Insulin resistance was defined as HOMA1-IR > 2.5 [21]. 

### 2.4. Definition of Chronic, Acute, and Acute-on-Chronic Hyperglycaemia

The presence of chronic hyperglycaemia in each patient was based on their medical history and HbA1c measurement. Euglycemia was defined as no prior DM diagnosis and HbA1c < 39 mmol/mol. Prediabetes was defined as no prior DM diagnosis and an HbA1c > 39 and <48 mmol/mol [22]. Known DM was defined as a prior DM diagnosis or treatment with glucose-lowering medication, regardless of the measured HbA1c level during admission, while the unknown DM definition was based on the absence of a prior DM diagnosis and an HbA1c ≥ 48 mmol/mol [11,22]. 

Acute hyperglycaemia was based on admission p-glucose, with p-glucose < 6.0 mmol/L classified as euglycaemia, p-glucose > 6.0 and <11 mmol/L as moderate, and p-glucose ≥ 11.0 mmol/L as severe hyperglycaemia [11]. 

The glycaemic gap, a measure of acute-on-chronic hyperglycaemia, was calculated as the difference between admission p-glucose and estimated average glucose derived from HbA1c and categorised as quartiles [10]. 

### 2.5. Outcome Measures, Exposures, and Confounders

The primary outcome was the change in CRP levels from admission until day 3 of hospitalisation. A follow-up of 3 days was selected because previous studies in CAP have indicated that a failure to observe a decline in CRP levels within the first 3–4 days of admission is associated with poor outcomes [6,23]. The secondary outcomes were peak CRP level and insulin resistance estimated with HOMA-IR. The chronic, acute and acute-on-chronic variables were the exposures when CRP was the outcome, while admission CPR was the exposure when HOMA-IR was the outcome. Systemic glucocorticoid treatment prescribed before or at hospital admission, regardless of dose, was considered a confounder in analyses of the CRP levels and HOMA-IR as dependent variables because glucocorticoids reduce CRP levels, increase insulin resistance and induce hyperglycaemia in patients with CAP [24]. 

### 2.6. Statistical Analyses

Continuous variables were skewed and thus summarised as a median with an interquartile range (IQR). Two-group comparisons of continuous variables were performed with the Wilcoxon rank sum test. Comparisons of continuous variables between more than two independent groups were conducted with Kruskal–Walli’s test, followed by Dunn’s post-hoc test in cases of statistically significant differences. Categorical variables were summarised as numbers and proportions and compared using chi-square tests. 

Repeated-measures linear mixed models based on a compound symmetry covariance pattern were used to model the association between chronic, acute, and acute-on-chronic hyperglycaemia variables as predictors and CRP levels at admission until day 3 as the dependent variable. Each predictor was evaluated as categorical and continuous variables in separate models. Chronic hyperglycaemia as a continuous variable was based on the HbA1c measurement. All models included time (i.e., hospitalisation day), fixed effects (i.e., covariates), and individual patients as random effects. Adjusted linear mixed models included age, sex, CURB-65 score, and glucocorticoid treatment as covariates. Separate linear mixed models with two-way interactions between time and each predictor were fitted to estimate differences in CRP levels at specific time points. 

Linear regression was used to model the association between admission CRP levels and HOMA-IR as the dependent variable. An unadjusted model and a model adjusted for age, sex, glucocorticoid treatment during hospitalisation, HbA1c, and BMI were fitted. CRP levels and HOMA-IR were logarithmically transformed in the regression analyses because of right-skewness and to fulfil model assumptions (in particular, normality of residuals) when evaluated as dependent variables. The reported exponentiated β-coefficients with 95% CI from the respective regression models represent percentage change. In cases of missing data, a complete case analysis was performed. The missingness pattern for each variable with missing observations is detailed in supplementary methods. 

R software with R version 4.0.3 (R Foundation for Statistical Computing, Vienna, Austria) was used for statistical analyses. The linear mixed model analyses were performed with the lme4 package. A *p*-value < 0.05 was considered statistically significant.

## 3. Results

### 3.1. Study Population

**Figure 1:**
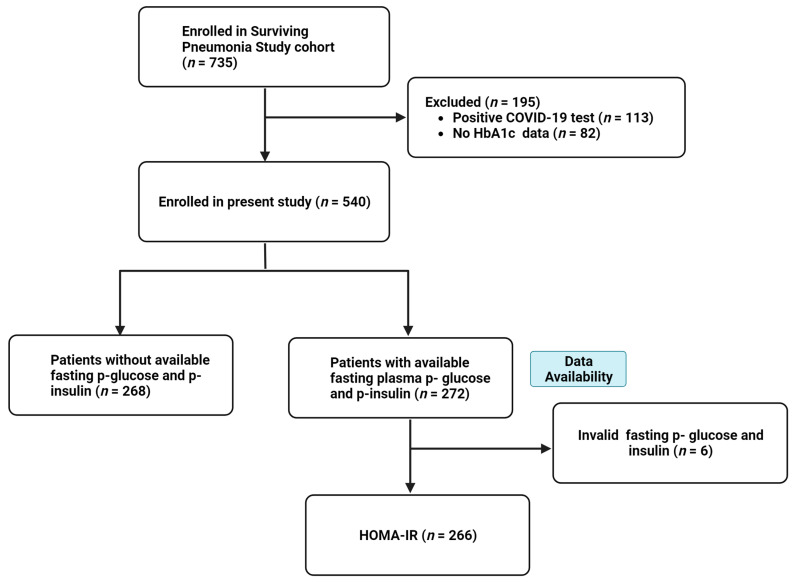
Flow chart of patient enrolment. Abbreviations: p, plasma; HOMA-IR, homeostasis model assessment for insulin resistance; COVID-19, coronavirus disease 2019.

Of the 735 patients enrolled in the Surviving Pneumonia Study cohort during the study period, 195 were excluded due to a positive COVID-19 test (*n* = 113) or a lack of available HbA1c measurement (*n* = 82), leaving 540 patients included in this study (Figure 1). Of these, 272 had available fasting plasma glucose and insulin samples, allowing for the estimation of HOMA-IR. However, samples from 6 patients were deemed invalid for HOMA-IR estimation due to exogenous insulin administration during the fasting period (Figure 1). Consequently, 540 patients were included in analyses with CRP as the outcome, while 266 were included in analyses with HOMA-IR as the outcome.

### 3.2. Patient Characteristics and Microbiological Findings

Patient characteristics are summarised in Table 1. Of the 540 patients, 48% were female, the median age was 74 (IQR 64, 81), and the median BMI was 26 kg/m^2^ (IQR 22, 30). The median Charlson comorbidity index was 4 (IQR 3–6), and 96 (18%) had a prior DM diagnosis, while 197 (36%) were diagnosed with chronic obstructive pulmonary disease (COPD). The frequencies of comorbidities are shown in Table A1, Appendix A. According to the CURB-65 score, 287 (53%), 183 (34%), and 70 (13%) patients had mild, moderate, or severe CAP, respectively. 

Respiratory samples from 194 patients were positive for at least 1 microbiological pathogen (Table A2). *Haemophilus influenzae* was the most isolated bacterium, while influenza A was the most isolated virus. Among patients with a bacterial infection, a Gram-negative bacterium was isolated from 106 patients, and a Gram-positive bacterium was isolated from 51 patients.

### 3.3. Patient Characteristics According to Chronic Hyperglycaemia

Chronic hyperglycaemia was observed in 315 (58%) patients, of whom 195 (36%) had prediabetes, 24 (4%) had unknown DM, and 96 (18%) had known DM (Table 1). There were no differences in age, sex distribution, or CURB-65 score between the chronic hyperglycaemia groups (Table 1). There were statistically significant differences in the Charlson comorbidity index, BMI, admission p-glucose and glycaemic gap between the chronic hyperglycaemia groups (Table 1). The Charlson comorbidity index was significantly different between patients with known DM and euglycaemia (*p* < 0.001) and between known DM and prediabetes (*p* = 0.04). The higher Charlson comorbidity index among patients with DM was driven by a higher proportion of cardiovascular diseases in these patients (Table A1). Among the 448 patients with a BMI measurement, there was a statistically significant difference in BMI between patients with DM and euglycaemia (*p* < 0.001). Moderate to severe acute hyperglycaemia was observed in 295 (55%) patients (Table 1). Admission p-glucose levels increased with worsening chronic hyperglycaemic status, with statistically significant differences between all groups except when comparing known and unknown DM. Regarding acute-on-chronic hyperglycaemia, there were statistically significant differences in the glycaemia gap between patients with known DM and prediabetes (*p* = 0.019) and between patients with prediabetes and euglycaemia (*p* = 0.009) (Table 1). 

### 3.4. Insulin Resistance

Among the 266 patients with a HOMA-IR estimation, 145 (55%) had insulin resistance (i.e., HOMA-IR > 2.5). Of the 230 patients without a prior DM diagnosis, 120 (52%) had insulin resistance (Table 1). The median HOMA-IR was 2.7 (1.7, 5.6) and increased with worsening chronic hyperglycaemia (Table 1). A comparison of HOMA-IR values across acute hyperglycaemia groups showed statistically significant differences between patients with euglycemia (HOMA-IR 2.6 [IQR 1.5, 4.5]) and those with severe acute hyperglycaemia (HOMA-IR 6.2 [IQR 2.4, 11.3], *p* = 0.034). No differences in HOMA-IR were observed across the glycaemic gap quartiles. The baseline characteristics of patients with and without a HOMA-IR estimation were similar (Table A3).

### 3.5. Glucocorticoids

Glucocorticoids were prescribed to 185 patients during hospitalisation, of whom 128 (69%) were diagnosed with COPD, and 25 (13%) continued with glucocorticoid treatment prescribed before admission. Patients with prediabetes were prescribed glucocorticoids more often than the other chronic hyperglycaemia groups (*p* = 0.034) (Table 1).

### 3.6. Association between Glycaemic Status, Glucocorticoids, and CRP Levels

**Figure 2:**
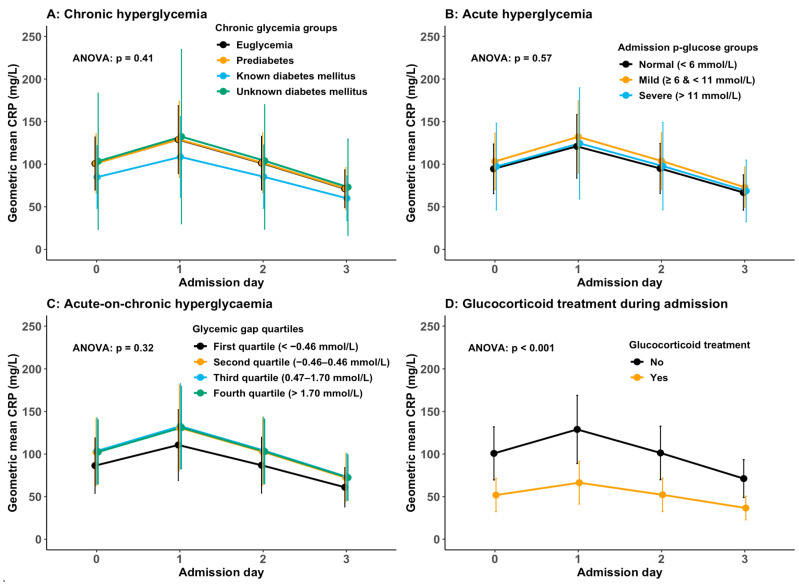
The figure shows the predicted values of geometric mean CRP with a 95% CI from admission until day 3 from linear, mixed models with age, sex, time, CURB-65 score, and corticosteroid treatment as fixed effects and patients as random effects. Predicted CRP values are stratified by chronic hyperglycaemia (**A**), acute hyperglycaemia (**B**), acute-on-chronic hyperglycaemia (**C**), and in-hospital glucocorticoid treatment (**D**). The *p*-value in each figure is from the analysis of variance of Type II multivariate Wald tests of fully adjusted linear mixed models for each predictor entered as a categorical variable (i.e., chronic hyperglycaemia groups, admission glucose groups, and glycaemic gap quartiles). The effect of glucocorticoid treatment was similar across the linear mixed models, and the *p*-value shown in (**D**) is from the model where chronic hyperglycaemia was the predictor.

Of the 540 patients included, 89 (16.5%) had missing CRP data on day 3. Patients without a day 3 CRP measurement had lower admission CRP levels compared to those with such a measurement (Table A4). Furthermore, patients with missing CRP data on day 3 had a median hospital stay of 2 days (IQR 1, 2), with 85% staying for 3 days or fewer. In contrast, patients with a day 3 CRP measurement had a median hospital stay of 6 days (IQR 4, 10), with only 11% staying 3 days or fewer (Table A4). As shown in Table 2 and Figure 2A–C, no association between chronic, acute, or acute-on-chronic hyperglycaemia and CRP levels was found, regardless of whether the chronic, acute, and acute-on-chronic variables were entered as continuous or categorical variables in the models. The effect of time was the same in all models (Table 2). 

No statistically significant interactions between the chronic, acute, or acute-on-chronic hyperglycaemia variables and time were found. As shown in Figure 2, the predicted CRP levels from the statistical models showed that CRP levels increased from admission to day 1 and declined afterwards. Admission CRP levels were similar, with overlapping 95% CIs between groups when stratified by chronic (Figure 2A), acute (Figure 2B), or acute-on-chronic hyperglycaemia (Figure 2C). The peak CRP levels were on day 1 and similar between groups when stratified by chronic (Figure 2A), acute (Figure 2B), or acute-on-chronic hyperglycaemia (Figure 2C). Glucocorticoid treatment had a similar effect across models and was associated with 48–49% lower CRP levels (Table 2, Figure 2D). 

### 3.7. Association between CRP at Admission and Insulin Resistance

The unadjusted analysis showed no association between admission CRP levels and HOMA-IR (β 1.04, 95% CI: 0.98–1.08, *p* = 0.24) (Table 3). The adjusted analysis showed a positive association between admission CRP levels and HOMA-IR (β 1.07, 95% CI: 1.01–1.14, *p* = 0.019), corresponding to a 7% (95% CI: 1–14%) increase in HOMA-IR for every 50 mg/L increase in admission CRP level (Table 3). Glucocorticoid treatment and increasing BMI were also associated with higher HOMA-IR (Table 3).

## 4. Discussion

The main finding of this study was that we found no association between chronic, acute, or acute-on-chronic hyperglycaemia and CRP levels from admission until day 3. In addition, we found that increasing admission CRP levels were associated with higher levels of insulin resistance.

Human in vivo and in vitro studies have shown that patients with DM have impaired secretion of inflammatory cytokines (e.g., IL-6) after stimulation with lipopolysaccharide (LPS), which is part of the outer cell wall of gram-negative bacteria [25,26,27]. These previous observations are particularly relevant as gram-negative bacteria are becoming the common causes of bacterial CAP [28,29], similar to the findings in our cohort. 

We hypothesised that chronic hyperglycaemia might affect CRP levels because systemic CRP levels progressively increase with progression from euglycaemia to prediabetes to DM in individuals without an infection secondary to low-grade infection [9]. Despite the previously described dysregulation of the IL-6-CRP axis among individuals with prediabetes or DM, we observed no association between prediabetes, known or unknown DM, and CRP levels during admission. Our findings are consistent with previous clinical studies concerning known DM [12,13,14]. Schuetz et al. [14] and Zeng et al. [13] found no differences in CRP levels, and Yende et al. [12] found no differences in the levels of the pro-inflammatory cytokines tumour necrosis factor-alpha, IL-6, and IL-10 during admission between patients with and without DM with CAP. 

Our study extends these previous findings to patients with prediabetes or unknown DM. Furthermore, Schuetz et al., Zeng et al., and Yende et al. based DM classification on a combination of self-reporting and medical history and had no HbA1c data [12,13,14]. Using a combination of medical history, self-reporting, and HbA1c data, we performed a detailed glycaemic characterisation and found a prevalence of prediabetes and unknown and known DM similar to a large, European multicentre cohort study of patients with CAP [11]. The detailed glycaemic characterisation of our cohort reduced the risk of misclassifying patients with unknown DM as having no DM. The availability of HbA1c data also enabled the investigation of the association between long-term glycaemic control and CRP levels. Patients prescribed glucocorticoids in our cohort had lower CRP levels, as previously described in CAP [24,30]. Although patients with prediabetes were prescribed glucocorticoids more often than the other chronic hyperglycaemia groups, adjusting for glucocorticoid therapy did not modify the association between chronic hyperglycaemia and CRP levels. Our study and the findings of others suggest that the impaired cytokine secretion associated with DM observed in preclinical studies does not affect the IL-6-CRP axis during a state of high-grade inflammation of a longer duration, like CAP. 

Similar to previous studies, more than half of the patients in our cohort had moderate to severe acute hyperglycaemia [10,11,31]. Human experimental studies have shown that inducing acute hyperglycaemia in healthy individuals increases IL-6 levels with or without LPS stimulation [32,33]. Despite these suggestive findings in healthy individuals, we did not observe an association between acute or acute-on-chronic hyperglycaemia and CRP levels during admission. Our findings contrast with those of Schuetz et al., who reported that moderate to severe acute hyperglycaemia was associated with higher CRP levels during hospitalisation than euglycaemia at admission among patients with CAP and no prior DM diagnosis [14]. 

Nevertheless, our study adds to the current literature by evaluating the impact of acute-on-chronic hyperglycaemia on CRP levels. Distinguishing between acute and acute-on-chronic hyperglycaemia is important, particularly in patients with known or unknown DM. Acute hyperglycaemia in patients with DM can reflect stress-induced hyperglycaemia or poor DM control with high baseline p-glucose levels, or both [15]. In addition, severe acute hyperglycaemia (i.e., admission p-glucose levels ≥ 11 mmol/L) has been associated with undiagnosed DM in patients with CAP and no prior DM diagnosis [11]. Therefore, differentiating between acute and acute-on-chronic hyperglycaemia enabled us to assess the effect of acute hyperglycaemia on CRP levels, most likely triggered by the stress associated with CAP. 

Insulin resistance is central to developing acute hyperglycaemia during acute illness [15,34]. The overall prevalence of insulin resistance was 55% and 52% among patients with no prior DM diagnosis. Furthermore, patients with severe acute hyperglycaemia at admission had higher HOMA-IR levels than patients with normal glucose levels. As far as we know, our study is the first to report an association between increasing admission CRP levels at admission and higher insulin resistance in patients with CAP. Furthermore, this association was independent of long-term glycaemic control, glucocorticoid treatment, and BMI. 

The small effect of admission CRP levels on HOMA-IR was surprising. Nonetheless, our findings are consistent with previous experimental studies in healthy individuals that showed insulin resistance increased in response to LPS infusion [34,35]. However, our findings differ from a smaller study that reported no correlation between systemic inflammation biomarkers (e.g., IL-6, IL-8, procalcitonin) and HOMA-IR in patients with severe infections, among whom 55% had pneumonia [36]. Furthermore, our analysis showed that glucocorticoid treatment and increasing BMI were associated with higher HOMA-IR levels. In addition to these identified factors, we hypothesise that other factors not measured in this study, such as stress hormones, body composition, and the extent of immobilisation due to bed rest before and during hospitalisation, might impact insulin resistance more than systemic inflammation, as measured by CRP, which is downstream of IL-6 signalling, in patients with CAP [5,15,37]. Whether the insulin resistance observed in our study persists after discharge or predicts future DM risk among patients with unknown DM is a relevant subject for future studies. 

The prospective design, standardised data collection, and detailed glycaemic characterisation are strengths of our study. In addition, the proportion of patients in our study with moderate to severe hyperglycaemia at admission, prediabetes, known DM, or unknown DM was comparable to prior studies of patients with CAP [10,11,31], strengthening the generalisability of our findings. Furthermore, by entering the chronic, acute, and acute-on-chronic hyperglycaemia variables as continuous variables in the models irrespective of previously established cut-off values for categorisation, we minimised information loss, increased statistical power, and improved the precision of the estimated effects.

However, our study has some limitations. First, attrition bias may have affected our results, as CRP measurements were not available on day 3 for 16.5% of the patients. Those with a missing CRP measurement on day 3 had lower CRP levels at admission, leading us to classify the pattern of missingness as ‘missing not at random. The most likely cause of missing CRP data on day 3 was early discharge. Therefore, we cannot exclude the possibility of bias in the reported estimates from analyses of the primary outcome [38]. Furthermore, estimates from these analyses might reflect findings from patients with a high initial systemic inflammatory response. Second, the absence of satiety data at the time of patient enrolment is another potential limitation. Consequently, we could not account for the confounding effects of recent food intake on glucose levels measured at admission. Third, the lack of adjustment for specific pathogens identified is a potential limitation, given that the host inflammatory response varies depending on the pathogen [39]. Nonetheless, a pathogen was not identified in 346 of the 540 patients, and among the 194 patients with an identified pathogen, the pathogens were diverse. This heterogeneity made it impractical to adjust for specific pathogens in our analysis. Fourth, although 17% of the patients had missing BMI data, which reduced the statistical power of the adjusted analysis of the association between admission CRP levels and HOMA-IR, we still found a significant association.

## 5. Conclusions

In conclusion, we found no association between CRP levels and acute, acute-on-chronic, or chronic hyperglycaemia. Secondly, we found a high prevalence of insulin resistance, and increasing CRP levels at admission were associated with higher insulin resistance. Our findings imply that the interpretation of CRP in the clinical management of CAP should not be influenced by chronic, acute, or acute-on-chronic glycaemic status.

## Figures and Tables

**Table 1:** Patient characteristics stratified by the presence of chronic hyperglycaemia ^1^.

Characteristic	*n*	Overall *n* = 540 ^2^	Euglycaemia *n* = 225 ^2^	Prediabetes *n* = 195 ^2^	Unknown Diabetes Mellitus *n* = 24 ^2^	Known Diabetes Mellitus *n* = 96 ^2^	*p*-Value ^3^
Demography							
Age (years)	540	74 (64, 81)	73 (62, 81)	74 (66, 81)	73 (71, 78)	73 (66, 79)	0.6
Female sex		257 (48%)	116 (52%)	89 (46%)	13 (54%)	39 (41%)	0.26
Anthropometry							
BMI^2^ (kg/m^2^)	448	26 (22, 30)	25 (21, 29)^#^	26 (22, 30)	27 (22, 29)	27 (25, 31)	<0.001
Comorbidity							
Charlson comorbidity index	540	4 (3–6)	4 (2–6) ^#^	4 (3–6) ^#^	5(4–7)	5 (4–7)	< 0.001
Chronic obstructive pulmonary disease	540	197 (36%)	72 (32%)	78 (40%)	9 (38%)	38 (40%)	0.33
Glucocorticoid treatment	540	185 (35%)	64 (29%)	82 (42%)	8 (33%)	31 (32%)	0.034
Disease severity							
CURB-65 score	540						0.10
Mild: 0–1		287 (53%)	135 (60%)	100 (51%)	9 (38%)	43 (45%)	
Moderate: 2		183 (34%)	67 (30%)	66 (34%)	10 (42%)	40 (42%)	
Severe: 3–5		70 (13%)	23 (10%)	29 (15%)	5 (21%)	13 (14%)	
Glycaemic parameters							
HbA1c (mmol/mol)	540	40 (37, 45)	36 (34, 38) ^§^	42 (40, 44) ^§^	51 (49, 53) ^§^	55 (48, 68) ^§^	<0.001
Admission glucose (mmol/L)	540	7.20 (6.22, 8.56)	6.62 (5.96, 7.57) ^#^	7.20 (6.31, 8.20) ^#^	8.54 (7.12, 9.19)	9.98 (7.62, 13.95)	<0.001
Acute hyperglycaemia groups							
Normal (<6 mmol/L)		245 (45%)	140 (62%)	85 (44%)	6 (25%)	14 (15%)	
Mild (6–11 mmol/L)		237 (44%)	81 (36%)	102 (52%)	15 (62%)	39 (41%)	
Severe (≥11.1 mmol/L)		58 (11%)	4 (1.8%)	8 (4.1%)	3 (12%)	43 (45%)	
Glycaemic Gap (mmol/L)	540	0.5 (−0.4; 1.8)	0.6 (−0.1; 1.8) ^#,¤^	0.3 (−0.6;1.2)	0.1 (−1.0; 0.9)	1.3 (−0.8; 3.8)	0.001
HOMA-IR	266	2.7 (1.7, 5.6)	2.4 (1.6, 3.9) ^#^	3.0 (1.9, 5.6)	2.8 (1.6, 4.3)	4.7 (2.4, 10.7)	0.013
HOMA-IR > 2.5	266	145 (55%)	52 (46%)	59 (58%)	9 (64%)	25 (69%)	0.049
Inflammatory parameters							
Admission CRP (mg/L)	540	109 (44, 181)	118 (46, 181)	116 (46, 187)	123 (55, 178)	84 (32, 156)	0.4
Peak CRP day 0–3 (mg/L)	540	151 (88, 225)	151 (101, 223)	155 (94, 245)	140 (93, 216)	149 (75, 204)	0.7

^1^ Definition: euglycaemia: HbA1c < 38 mmol/mol and no prior DM diagnosis; prediabetes: HbA1c > 38 and <48 mmol/mol and no prior DM diagnosis; unknown DM: HbA1c ≥ 48 mmol/mol and no prior DM diagnosis. ^2^ Continuous variables are summarised as the median (IQR); categorical variables are summarised as *n* (%). ^3^ Kruskal–Wallis rank sum test; Pearson’s Chi-squared test; Fisher’s exact test. ^#^ Statistically significant difference compared to patients with known DM. ^¤^ Statistically significant difference compared to patients with prediabetes. ^§^ Statistically significant differences between all groups. Abbreviations: BMI, body mass index; CRP, c-reactive protein; CURB-65 score is based on 5 variables: Confusion, Urea > 7 mmol/L, Respiratory rate ≥ 30 breaths/minute, systolic Blood pressure < 90 mmHg or diastolic Blood pressure ≤ 60 mmHg and age ≥ 65 years; HOMA-IR, homeostasis model assessment of insulin resistance; SD, standard deviation; IQR, interquartile range.

**Table 2:** The association between chronic, acute, and acute-on-chronic hyperglycaemia and CRP levels.

	Model 1 ^1^			Model 2 ^2^		
Predictors	Estimates (β)	95% CI	*p*-Value	Estimates (β)	95% CI	*p*-Value
^3^ Effect of time						
Admission day (reference)						
Day 1	1.28	1.20–1.36	<0.001	1.28	1.20–1.36	
Day 2	1.01	0.95–1.07	0.87	1.01	0.95–1.07	0.87
Day 3	0.71	0.66–0.75	<0.001	0.71	0.66–0.75	
Chronic hyperglycemia models						
HbA1c (Δ1 mmol/mol)	1.00	0.99–1.01	0.87	1.00	0.99–1.01	0.73
Glucocorticoid treatment (no)—reference						
Glucocorticoid treatment (yes)				0.52	0.44–0.61	< 0.001
Chronic glycaemia groups ^4^						
Euglycaemia (reference)						
Known DM	0.84	0.67–1.06	0.13	0.84	0.67–1.05	0.13
Prediabetes	0.92	0.76–1.10	0.36	1.00	0.84–1.20	0.98
Unknown DM	1.01	0.67–1.51	0.98	1.03	0.70–1.51	0.90
Glucocorticoid treatment (no)—reference						
Glucocorticoid treatment (yes)				0.51	0.44–0.61	<0.001
Acute hyperglycaemia models						
Admission p-glucose (Δ1 mmol/L)	1.01	0.99–1.04	0.34	1.01	0.99–1.04	0.28
Glucocorticoid treatment (no)—reference						
Glucocorticoid treatment (yes)				0.52	0.44–0.61	<0.001
Admission p-glucose groups						
p-glucose <6 mmol/l (reference)						
p-glucose > 6 & <11 mmol/L	1.04	0.87–1.23	0.68	1.09	0.93–1.29	0.30
p-glucose > 11 mmol/L	0.97	0.73–1.27	0.80	1.03	0.79–1.34	0.81
Glucocorticoid treatment (no)—reference						
Glucocorticoid treatment (yes)				0.51	0.44–0.60	<0.001
Acute-on-chronic hyperglycaemia models						
Glycaemic gap (Δ1 mmol/L)	1.02	0.99–1.06	0.17	1.03	1.00–1.06	0.10
Glucocorticoid treatment (no)—reference						
Glucocorticoid treatment (yes)				0.51	0.44–0.60	<0.001
Glycaemic gap quartiles ^5^						
1st quartile (reference)						
2nd quartile	1.09	0.86–1.37	0.49	1.18	0.95–1.48	0.14
3rd quartile	1.16	0.92–1.46	0.22	1.20	0.96–1.50	0.11
4th quartiles	1.16	0.92–1.46	0.20	1.19	0.95–1.47	0.12
Glucocorticoid treatment (no)—reference						
Glucocorticoid treatment (yes)				0.51	0.44–0.60	< 0.001

Data are from repeated linear mixed model analysis and are reported as the percentage change in regression coefficient (β) after back transformation. ^1^ Model 1: unadjusted. ^2^ Model 2: adjusted for age, sex, prescribed glucocorticoids in hospital and CURB-65 score. ^3^ Effect of time on CRP levels was the same in all models. ^4^ Definitions: euglycaemia: HbA1c < 38 mmol/mol and no prior DM diagnosis; prediabetes: HbA1c > 38 & <48 mmol/mol & no prior DM diagnosis; unknown DM: HbA1c ≥ 48 mmol/mol and no prior DM diagnosis. Abbreviations: DM, diabetes mellitus, CI, confidence interval; p, *p*-value; p-glucose, plasma glucose. ^5^ Glycaemic gap quartile ranges: 1st quartile: <−0.46 mmol/L; 2nd quartile: −0.46–0.46 mmol/L; 3rd quartile: 0.47–1.70 mmol/L; 4th quartile: >1.70 mmol/L.

**Table 3:** Association between CRP levels at admission and HOMA-IR.

	Unadjusted Model			Adjusted Model		
Predictors	Estimates (β)	95% CI	*p*-Value	Estimates (β)	95% CI	*p*-Value
CRP at admission (Δ50 mg/L)	1.03	0.98–1.08	0.29	1.07	1.01–1.14	0.019
Age (Δ1 year)				0.99	0.98–1.00	0.19
Sex (female)—reference						
Sex (male)				1.11	0.88–1.39	0.39
HbA1c (Δ1 mmol/mol)				1.00	0.99–1.01	0.99
Glucocorticoid treatment (no)—reference						
Glucocorticoid treatment (yes)				1.65	1.29–2.12	<0.001
BMI (Δ1 kg/m^2^)				1.06	1.05–1.09	<0.001

Data are from a linear regression analysis reported as the percentage change in regression coefficient (β) after back transformation. Abbreviations: Δ, delta; HOMA-IR, homeostasis model assessment of insulin resistance; HbA1c, glycated haemoglobin; BMI, body mass index.

## Data Availability

The datasets used for the current study are not publicly available. However, relevant pseudonymised data can be accessed upon a reasonable request.

## Appendix A

### Appendix A.1. Supplementary Statistical Methods

An indicator variable for missingness for each variable with missing data was coded as a binary [dummy] variable. Logistic regression was used to investigate the association between the indicator variable for missingness as the dependent variable and the other variables included in the primary analysis as predictors. In addition, linear regression was used to investigate whether variables significantly associated with the indicator variable for missingness in the logistic regression analyses were associated with the observed values of the continuous variables with missing data as the dependent variable. Eighty-nine (16.5%) patients had missing CRP data on day 3. The missingness pattern for CPR on day 3 was assumed to be “missing not at random” since it was associated with lower admission CRP levels. Ninety-two patients (17%) had no BMI measurement during admission. The missingness pattern for BMI was classified as “missing at random” because it was associated with a moderate to severe CURB-65 score. Further, there was no difference in BMI between CURB-65 score groups among patients with observed BMI.

### Appendix A.2. Supplementary Tables

**Table A1:** Frequencies of comorbidities included in the Charlson comorbidity index.

Characteristic	Overall, *n* = 540 ^1^	Euglycaemia ^3^, *n* = 225 ^1^	Prediabetes ^3^, *n* = 195 ^1^	Unknown Diabetes Mellitus ^3^, *n* = 24 ^1^	Known Diabetes Mellitus ^3^, *n* = 96 ^1^	*p*-Value *^2^*
Charlson comorbidity index	4 (3, 6)	4 (2, 6)	4 (3, 6)	5 (3.75, 7.00)	5.00 (4.00, 7.00)	<0.001
History of myocardial infarction	53 (9.8%)	11 (4.9%)	25 (13%)	3 (12%)	14 (15%)	0.006
Heart failure	87 (16%)	26 (12%)	32 (16%)	6 (25%)	23 (24%)	0.023
Peripheral vascular disease	22 (4.1%)	4 (1.8%)	6 (3.1%)	3 (12%)	9 (9.4%)	0.003
Previous stroke	78 (14%)	27 (12%)	27 (14%)	5 (21%)	19 (20%)	0.2
Dementia	12 (2.2%)	6 (2.7%)	5 (2.6%)	0 (0%)	1 (1.0%)	0.9
COPD	197 (36%)	72 (32%)	78 (40%)	9 (38%)	38 (40%)	0.3
Connective tissue disease	32 (5.9%)	19 (8.4%)	8 (4.1%)	1 (4.2%)	4 (4.2%)	0.2
History of peptic ulcer disease	46 (8.5%)	12 (5.3%)	23 (12%)	2 (8.3%)	9 (9.4%)	0.10
Liver disease	13 (2.4%)	6 (2.7%)	4 (2.1%)	0 (0%)	3 (3.1%)	0.9
Hemiplegia	14 (2.6%)	6 (2.7%)	5 (2.6%)	0 (0%)	3 (3.1%)	>0.9
Moderate to severe chronic kidney disease	17 (3.1%)	6 (2.7%)	6 (3.1%)	0 (0%)	5 (5.2%)	0.6
Cancer	104 (19%)	46 (20%)	39 (20%)	8 (33%)	11 (11%)	0.064
HIV	0 (0%)	0 (0%)	0 (0%)	0 (0%)	0 (0%)	>0.9

^1^ Median (IQR); *n* (%). ^2^ Kruskal-Wallis rank sum test; Pearson’s Chi-squared test; Fisher’s exact test. ^3^ Definitions: euglycaemia: HbA1c < 38 mmol/mol and no prior DM diagnosis; prediabetes: HbA1c > 38 & <48 mmol/mol & no prior DM diagnosis; unknown DM: HbA1c ≥ 48 mmol/mol and no prior DM diagnosis. Abbreviations: COPD, chronic obstructive pulmonary disease; HIV, human immunodeficiency virus.

**Table A2:** Microbial aetiology.

Bacterial Aetiology	*n*
Other gram-negative bacterium	46
*Haemophilus influenzae*	27
*Streptococcus pneumoniae*	21
*Legionella pneumophila*	9
*Staphyloccocus aureus*	9
*M. pneumoniae*	5
*Pseudomonas aeruginosa*	5
*Streptococcus* spp.	4
*Moraxella catarrhalis*	3
*P. aeruginosa* & other gram-negative bacterium	2
*S. pneumoniae* & *H. influenzae*	2
*H. influenzae* & *L. pneumophila*	1
*H. influenzae* & *M. catarrhalis*	1
*H. influenzae* & other gram-negative bacterium	1
*H. influenzae* & *S. aureus*	1
*Legionella.* & *S. aureus* & other gram-negative bacterium	1
*S. aureus* & *P. aeruginosa* & *M. catarrhalis* & other gram-negative bacterium	1
*S. pneumoniae* & *S. aureus*	1
*S. aureus* & *Streptococcus* spp.	1
*Streptococcus* spp. & *S. aureus* & other gram-negative bacterium	1
Mixed bacterial and viral aetiology	
*S. pneumoniae* & rhinovirus	3
*S. pneumoniae* & parainfluenza A virus	2
*H. influenzae* & human metapneumovirus	1
*H. influenzae* & influenza A virus	1
Other gram-negative bacterium & influenza A virus	1
*P. aeruginosa* & influenza A virus	1
*P. aeruginosa* & respiratory syncytial virus	1
*S. aureus* & influenza A virus	1
*S. pneumoniae* & human metapneumovirus	1
*Streptococcus* spp. & respiratory syncytial virus	1
*S. aureus* & influenza A virus	1
Viral aetiology	
Influenza A virus	23
Respiratory syncytial virus	5
Human metapneumovirus	4
Rhinovirus	3
Parainfluenza A virus	2
Adenovirus	1

Other gram-negative bacteria encompass isolation of *Escherichia coli*, *Klebsiella pneumoniae*, *K. oxytoca*, *Enterobacter* spp., *Stenotrophomonas maltophilia*, *Eikenella corrodens*, *Neisseria meningitidis*, *Proteus* spp. or *P. mirabilis*.

**Table A3:** Patient characteristics according to the availability of a HOMA-IR measurement.

Characteristic	No HOMA-IR ^1^ Available (*n* = 274)	HOMA-IR Available ^1^ (*n* = 266)	*p*-Value ^2^
Demography			
Age, years	73 (63, 80)	75 (66, 82)	0.051
Sex, female	130 (47%)	127 (48%)	0.9
Comorbidities			
Charlson comorbidity index	4 (3, 6)	4 (3, 6)	0.8
Chronic hyperglycemia groups ^3^			0.072
Euglycaemia	111 (41%)	114 (43%)	
Prediabetes	93 (34%)	102 (38%)	
Unknown diabetes mellitus	10 (3.6%)	14 (5.3%)	
Known diabetes mellitus	60 (22%)	36 (14%)	
Chronic obstructive pulmonary disease	96 (35%)	101 (38%)	0.5
Glucocorticoid treatment	87 (32%)	98 (37%)	0.2
Disease severity			
CURB-65 score			0.8
Mild (0–1)	149 (54%)	138 (52%)	
Moderate (2)	90 (33%)	93 (35%)	
Severe (3–5)	35 (13%)	35 (13%)	
Glycaemic parameters			
HOMA-IR	2.7 (1.7, 5.6)	-	
HbA1c (mmol/mol)	40 (36, 46)	40 (37, 44)	0.5
Admission glucose (mmol/L)	7.2 (6.1, 8.5)	7.2 (6.3, 8.6)	0.4
Glycaemic Gap (mmol/l)	0.5 (−0.6, 1.8)	0.5 (−0.3, 1.8)	0.3
BMI kg/m^2^	26 (23, 30)	26 (22, 29)	0.6
Inflammatory parameters			
Admission CRP (mg/l)	99 (35, 171)	124 (52, 187)	0.033

^1^ Continuous are summarised with median (IQR); categorical variables are summarised with *n* (%). ^2^ Wilcoxon rank sum test for continuous variables; Pearson’s Chi-squared test for categorical variables. ^3^ Definitions: euglycaemia: HbA1c < 38 mmol/mol and no prior DM diagnosis; prediabetes: HbA1c > 38 & <48 mmol/mol & no prior DM diagnosis; unknown DM: HbA1c ≥ 48 mmol/mol and no prior DM diagnosis. Abbreviations: BMI, body mass index; DM, diabetes mellitus, CRP, c-reactive protein; CURB-65 score is based on 5 variables: Confusion, Urea >7 mmol/L, Respiratory rate ≥30 breaths/minute, systolic Blood pressure <90 mmHg or diastolic Blood pressure ≤60 mmHg and Age ≥65 years; HOMA-IR, homeostasis model assessment of insulin resistance; SD, standard deviation; IQR, interquartile range.

**Table A4:** Patient characteristics according to the availability of a day 3 CRP measurement.

Characteristic	No day 3 CRP Measurement, *n* = 89 ^1^	Day 3 CRP Measurement Available, *n* = 451 ^1^	*p*-Value ^2^
Age (years)	73 (60, 79)	74 (65, 81)	0.2
Sex (female)	56%	46%	0.076
Charlson comorbidity index	4.0 (2.8, 6.00)	5.0 (3.0, 6.0)	0.034
CURB-65 score, severity			0.094
Mild (0–1)	56 (63%)	231 (51%)	
Moderate (2)	26 (29%)	157 (35%)	
Severe (3–5)	7 (7.9%)	63 (14%)	
Admission CRP (mg/l)	57 (18, 118)	126 (51, 188)	<0.001
Length of stay (days)	2 (1, 2)	6 (4, 10)	<0.001
Length of stay ≤ 3 days	85%	11%	<0.001
Died in-hospital	6.7%	6.9%	>0.9

^1^ Continuous are summarised with median (IQR); categorical variables are summarised with %. ^2^ Wilcoxon rank sum test for continuous variables; Pearson’s Chi-squared test for categorical variables. Abbreviations: CRP, C-reactive protein.
